# A Method for Fast Au-Sn Bonding at Low Temperature Using Thermal Gradient

**DOI:** 10.3390/mi14122242

**Published:** 2023-12-15

**Authors:** Wenchao Wang, Ziyu Liu, Delong Qiu, Zhiyuan Zhu, Na Yan, Shijin Ding, David Wei Zhang

**Affiliations:** 1 State Key Laboratory of Integrated Chips and Systems, School of Microelectronics, Fudan University, Shanghai 200433, China; wangwenchao@fudan-js.org.cn (W.W.); yanna@fudan.edu.cn (N.Y.); sjding@fudan-js.org.cn (S.D.); dwzhang@fudan.edu.cn (D.W.Z.); 2 Jiashan Fudan Institute, Jiaxing 314100, China; qiudelong@fudan-js.org.cn; 3 School of Electronic Information Engineering, Southwest University, Chongqing 404100, China; zyuanzhu@swu.edu.cn; 4 National Key Laboratory of Materials for Integrated Circuits, Shanghai Institute of Microsystem and Information Technology, Chinese Academy of Sciences, 865 Changning Road, Shanghai 200050, China

**Keywords:** flip chip, Au-Sn, solid-state diffusion (SSD), thermal gradient bonding (TGB), intermetallic compound (IMC)

## Abstract

Flip chip bonding technology on gold–tin (Au-Sn) microbumps for MEMS (Micro Electro Mechanical Systems) and 3D packaging is becoming increasingly important in the electronics industry. The main advantages of Au-Sn microbumps are a low electrical resistance, high electrical reliability, and fine pitch. However, the bonding temperature is relatively high, and the forming mechanism of an intermetallic compound (IMC) is complicated. In this study, Au-Sn solid-state diffusion (SSD) bonding is performed using the thermal gradient bonding (TGB) method, which lowers bonding temperature and gains high bonding strength in a short time. Firstly, Au-Sn microbumps with a low roughness are prepared by using an optimized process. Then, Au-Sn bonding parameters including bonding temperature, bonding time, and bonding pressure are optimized to obtain a higher bonding quality. The shear strength of 23.898 MPa is obtained when bonding in the HCOOH environment for 10 min at the gradient temperature of 150 °C/250 °C with a bonding pressure of more than 10 MPa. The IMC of Au-Sn is found to be Au-Sn and Au_5_Sn. The effect of annealing time on the IMC is also investigated. More and more Au_5_Sn is generated with an increase in annealing time, and Au_5_Sn is formed after Sn is depleted. Finally, the effect of annealing time on the IMC is verified by using finite element simulation, and the bonding strength of IMC was found to be higher when the bonding temperature is 150 °C at the cold side and 250 °C at the hot side. The temperature in the bonding area can reach 200 °C, which proves that the Au-Sn bonding process is solid-state diffusion because the temperature gradient reaches 2500 °C/cm.

## 1. Introduction

In recent years, the electronics industry has witnessed a surging demand for microbump flip-chip interconnections, driven by the miniaturization and portability demand of electronic products [1,2]. This brings several challenges such as smaller pitch bonding and higher overall reliability [3,4]. Au-Sn microbump bonding emerges as a prominent candidate due to Au-Sn intermetallic compounds (IMC) offering superior mechanical properties, lower electrical conductivity, and higher thermal conductivity [5,6,7]. Bonding techniques for Au-Sn microbumps encompass eutectic bonding and solid–liquid interdiffusion (SLID) bonding. Eutectic bonding technology is widely used due to its low bonding pressure and capacity for high-density integration [8,9,10,11,12,13].

Traditionally, the Au-Sn eutectic bonding process proceeds at the temperature range of 280 °C–350 °C [14,15], involving solid–liquid diffusion bonding or instantaneous liquid-phase bonding. However, this method carries the risk of Sn extrusion, leading to potential short-circuits [16,17]. Additionally, the high bonding temperature engenders significant residual thermal stress, weakening the module and device reliability [18]. Lower bonding temperatures prolong bonding times, decrease productivity, escalate process costs, and introduce bonding quality issues, which limits applicability in multi-chip interconnection. High temperatures increase Sn oxidation, necessitating special environments like H_2_, HCOOH, or vacuum during bonding [19].

To circumvent the challenges posed by high temperatures, solid-state diffusion bonding techniques at lower temperatures have gained attraction. These techniques typically operate below the melting point of Sn (231.89 °C) [20]. Noteworthy research by the University of Tokyo explores low-temperature solid-state Au-Sn bonding, leveraging the surface-activated bonding (SAB) method to bond 30 µm pitch Au-Sn microbumps in ambient air SAB [21]. Nevertheless, certain aspects related to reliability remain to be elucidated. In a series of studies, a team from National Taiwan University discovered a temperature gradient bonding method to expedite the diffusion between Cu-Sn atoms, reducing bonding time and enhancing production efficiency [22]. To mitigate surface oxide issues on Sn bumps, the team from Osaka University achieved high-quality, low-temperature solid-state Cu-Sn bonding by introducing HCOOH vapor under specific conditions (200 °C, 20 MPa, and 20 min). Remarkably, shear strengths of samples bonded in an HCOOH environment surpassed those bonded in an N_2_ environment [23,24]. The team at Tsinghua University investigated the low-temperature Cu/Sn/Cu solid-state diffusion (SSD) bonding technique by treating the microbumps with Ar (5% H_2_) plasma and obtained an average bond strength of 5 MPa after bonding at 200 °C for 60 min at a bonding pressure of 6.7 MPa [25]. Few studies about Au-Sn bonding at lower temperatures are found.

This study harnesses lower-temperature, high-quality, and narrow-pitch Au-Sn bonding by the mechanism of solid-state diffusion. To lower the bonding temperature, Au-Sn microbump quality is first optimized by the electroplating and evaporative deposition processes. A pretreatment method is employed to eliminate surface oxides from the microbumps before bonding. Subsequently, a temperature gradient method in an HCOOH environment is applied and validated in the Au-Sn microbump bonding with a pitch of 20 µm. Finite element simulations corroborate that the bonding process involves solid-state diffusion in the temperature gradient bonding. Through the integration of simulation and experiment, the objective is to provide insights into the rapid low-temperature Au-Sn bonding process and optimize bonding parameters for prospective microelectronic packaging applications.

## 2. Materials and Methods

### 2.1. Preparation of Microbump and Bonded Chip

**Figure 1:**
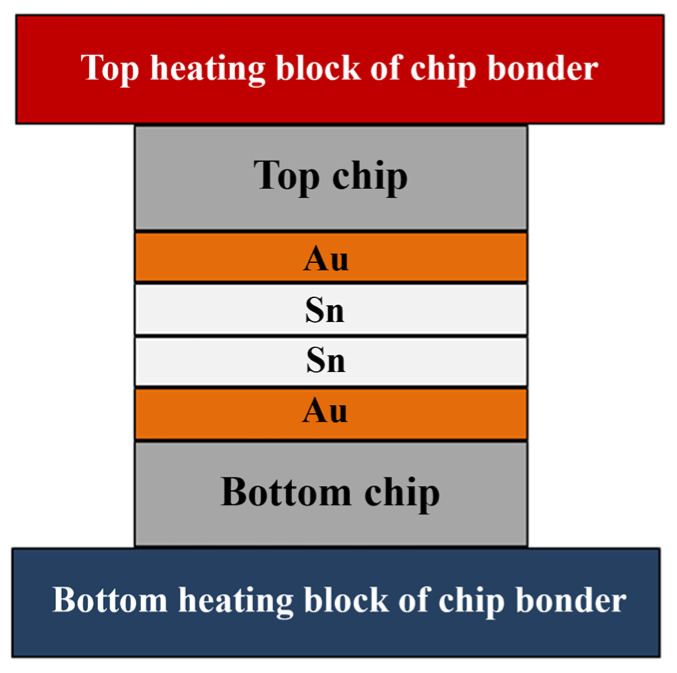
Temperature gradient bonding (TGB) structure.

The bonding structure of Au-Sn microbumps is illustrated in Figure 1, featuring the Au/Sn-Sn/Au sandwich structure of the bumps. The Au bumps are dimensionally congruent with the Sn bumps, with a size of 10 × 10 µm. Heat conduction through the top and bottom ceramic heating blocks facilitates isothermal heating. Alternatively, a temperature gradient can be formed by maintaining different temperatures at the top and bottom heating blocks.

**Figure 2:**
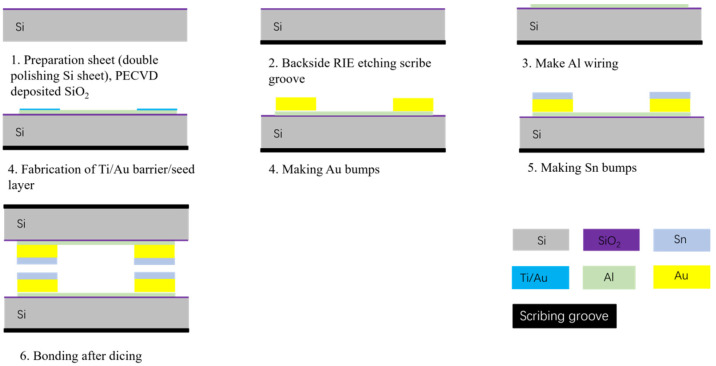
Bonded chip manufacturing flowchart.

In this study, the process was initiated by creating scribe grooves and alignment marks on the backside of a 550 mm thick double-throw silicon oxide wafer by Reactive ion etching. This step is essential for facilitating subsequent alignment bonding. Then, an Al metal layer with a thickness of 1 µm was deposited by sputtering and etched by the wetting process. The Au/Sn microbumps were then fabricated by an electroplating and evaporation process, respectively. The Au/Sn microbumps had a thickness of 4 µm/2 µm. Subsequently, wafers were sliced into a bottom bonding chip with 1.2 cm × 1.2 cm and a top bonding chip with 1 cm × 1 cm. These chips were placed on the bonding equipment for pre-bonding experiments, as illustrated in Figure 2.

The bonding parameter optimization was conducted by changing the bonding time, bonding pressure, and bonding temperature. The bonding was carried out using an FC150 (SUSS MicroTec is located in Garching, Germany). After bonding, the shear strength of interconnecting bumps on the bonded chips was assessed on a shear force tester. To observe the IMC, the cross-sectional sample was prepared by an EcoMet30 (Buehler is based in Lake Bluff) grinding and polishing machine (diamond polishing disk roughness ~0.05 µm) and a Leica EM TIC 3X (Leica Microsystems in Vizsla, Germany) triple-ion beam dicing machine. Finally, we characterized the microstructure of the IMC through scanning electron microscopy (GeminiSEM 300, ZEISS in Oberkochen, Germany) combined with chemical analysis by an EDS energy spectrometer.

### 2.2. Bonding Temperature Distribution Simulation

To reveal the temperature distribution at the bonding interface and uncover the thermal diffusion mechanism, finite element simulation was investigated using COMSOL in both isothermal bonding and temperature gradient bonding [26,27,28,29]. The bonding model is the same as the bonding structure in Figure 1. For heat transfer analysis, engineering problems involving heat flow rate and temperature parameters in the structure are typically assessed. The nonlinear flow equation is usually expressed as below [30]:

$$\left\{Q\right\}=\left[C\right]\frac{d\left\{T\right\}}{dt}+\left[K\right]\left\{T\right\}$$

where, K represents the conduction matrix encompassing the heat conduction, radiation rate, convection coefficient, and shape coefficient. {T} signifies the temperature vector for each computing node, [C] stands for the fixed specific heat capacity matrix, and {Q} represents the heat flow rate load vector. By examining changes in heat flux caused by variations in the system’s temperature field over time, the temperature field was analyzed at a constant temperature, which was maintained during steady-state conditions. At this stage, the system attained equilibrium, with heat inflow and generation equaling heat outflow. Under these conditions, the heat flow equation is simplified as {Q} = [K]{T}.

Heat transfer takes place through three mechanisms: heat convection, heat radiation, and heat diffusion. Thermal diffusion, particularly involved in this study, means the transfer of heat from regions of higher temperature to those with lower temperature due to a temperature gradient. This diffusion process is controlled by Fourier’s law [31]:

$$q\underset{q}{\to }=\frac{Q}{A}=-k\nabla T=\frac{\delta t}{\delta x}\underset{l}{\to }+\frac{\delta t}{\delta y}\underset{j}{\to }+\frac{\delta t}{\delta z}\underset{k}{\to }$$

where k represents thermal conductivity (W/(m·K)), and A is the thermal conductivity area (m^2^).

**Figure 3:**
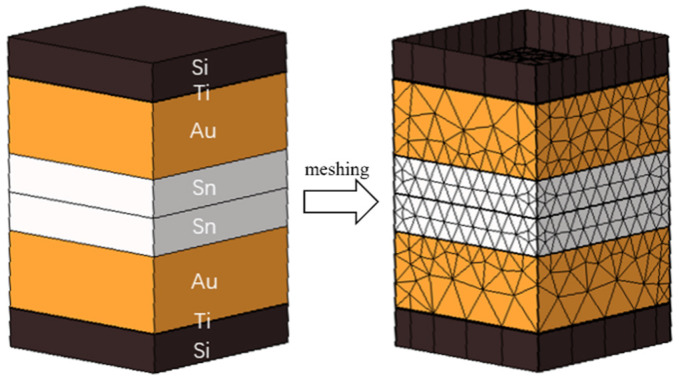
Locally bonded model and meshed model.

As depicted in Figure 3, the simulation modeling is based on the actual size of the microbumps. The silicon oxide layer, seed layer, and Al wiring layer were excluded because the focus was on simulating the heating device in the bonding process by applying temperatures at the ends of the top and bottom Si sheets. This study employed mesh delineation to create the model.

## 3. Results and Discussion

In this work, several bonding parameters were firstly proposed to optimize a set of bonding parameters under TGB bonding technology. Then, the comparison between thermal gradient bonding and isothermal bonding was performed to derive the underlying reason for the higher strength. Then, the finite element simulation method was further proposed to verify the conclusion and confirm the TGB bonding belonging to solid diffusion. At last, the IMC evolution was investigated to uncover the stable IMC phase, which is important when applied for high-power device interconnection.

### 3.1. Bonding Parameter Optimization

**Figure 4:**
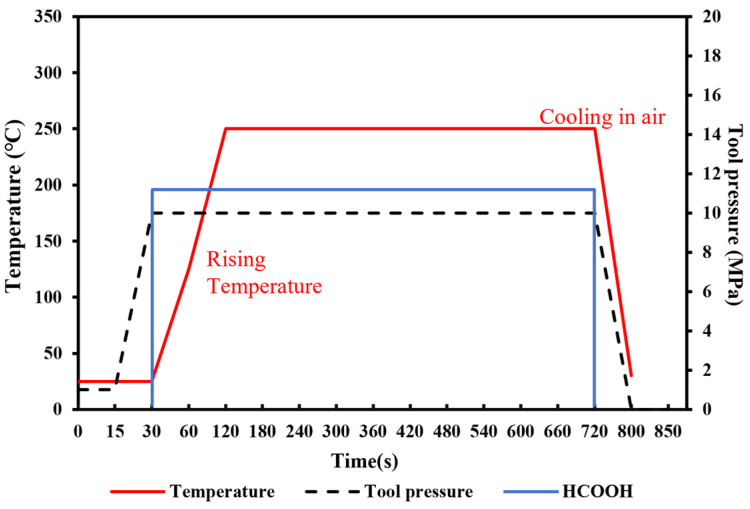
Schematic diagram of original bonding parameters with gradient temperature of 150 °C/250 °C under the pressure of 10 MPa.

**Figure 5:**
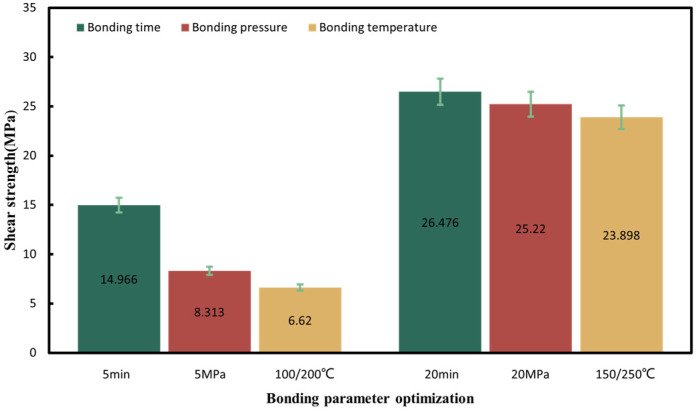
Shear strength summary for all the bonding parameters in the gradient bonding.

To achieve the robust chip-scale Au-Sn bonding with a low temperature, short time and high bonding strength, preliminary tuning of the pre-bonding parameters, including the bonding time, temperature and pressure, was performed under the HCOOH treatment condition. Several sets of parameters were tested, including the bonding time of 5, 10, and 20 min, the bonding pressures of 5, 10, and 20 MPa, and the gradient bonding temperature of 100 °C/200 °C and 150 °C/250 °C. The wafer pairs were bonded in an FC150 bonder from Suss MicroTech. Figure 4 shows the set of bonding parameter profiles with the formic acid, bonding temperature and pressure. Then, experiment parameters above are designed based on modifying one parameter from the set of bonding parameters in Figure 4. When one parameter was modified, the other bonding parameters were kept unchanged. Figure 5 provides the shear strength for all the bonding samples. It was found that the condition of 150 °C/250 °C with a pressure of 10 MPa for 10 min was the most optimal based on its higher bonding strength, shorter bonding time and lower bonding pressure. Later, the optimized samples were anlyzed to reveal the reason why the bonding can be succeeded.

### 3.2. IMC Comparison of Thermal Gradient Bonding and Isothermal Bonding

**Figure 6:**
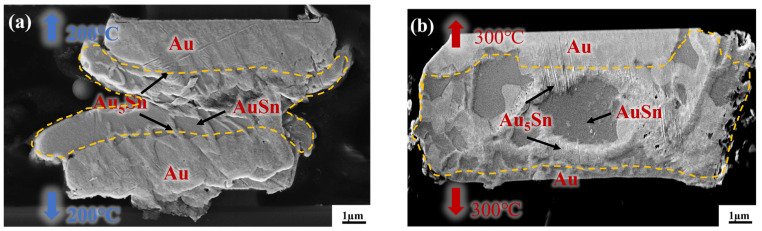
Cross-section observation of the bump IMC at the bonding temperature of (**a**) 200 °C and (**b**) 300 °C.

To reveal the underlying reason for the high strength of the gradient bonding, IMC comparison was first conducted between the isothermal bonding and thermal gradient bonding. Figure 6a,b depict the cross-sectional morphology of Au-Sn/Sn-Au bumps after the isothermal bonding at uniform temperatures of 200 °C and 300 °C for 10 min, respectively. Figure 6a demonstrates there is a small amount of Au_5_Sn and Au-Sn phases at the interface based on the energy-dispersive spectroscopy. However, a substantial quantity of metallic Sn remained unconsumed for the IMC formation. This illustrates that the IMC, at the temperature of 200 °C for 10 min, forms quite slowly and the temperature alone is insufficient for inducing high growth rates. Then, the isothermal bonding temperature rose to 300 °C to increase the IMC growth rate. Figure 6b presents the cross-sectional morphology of IMC at the bonding temperature of 300 °C for 10 min. It is evident that most of the metallic Sn within the bonding area was almost consumed within 10 min, resulting in the formation of two phases: Au_5_Sn and Au-Sn. The rationale behind this observation is that the bonding temperature of 300 °C surpasses the melting point of metallic Sn. Consequently, Sn exists in a molten state during the bonding process, facilitating diffusion. However, Sn overflow will happen and leads to larger bonding interface size. Additionally, high temperatures may pose the risk of device damage.

**Figure 7:**
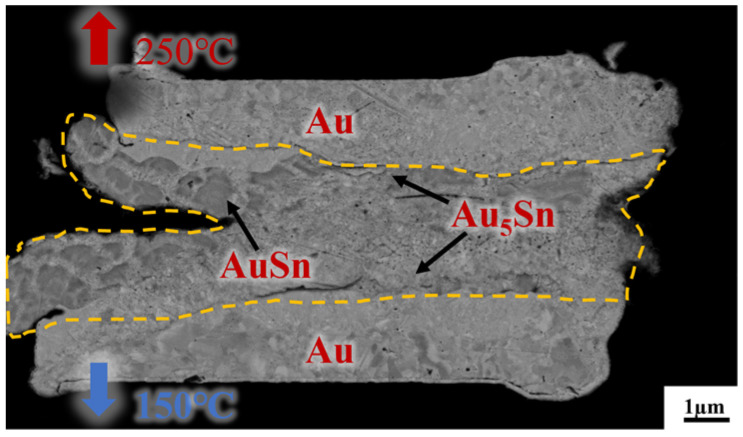
Cross-section observation of the bump IMC at the bonding temperature of 150 °C/250 °C, with a bonding pressure of 10 MPa and bonding time of 10 min.

Figure 7 presents a cross-sectional view of Au/Sn-Sn/Au bumps at the thermal gradient bonding (TGB) with the temperature of 250 °C at the top heating block and 150 °C at the bottom heating block for 10 min. It shows the IMC thickness was larger than that at the isothermal temperature of 200 °C and resulted in more Au_5_Sn and Au-Sn phases in the IMC region. This means the bonding process consumed a significant portion of metallic Sn. This particular configuration also proves that the high bonding strength (23.898 MPa in Figure 5) comes from the thick IMC for TGB bonding with a temperature gradient of 150 and 250 °C. The high strength can satisfy the subsequent chip stacking, credited to the abundant Au_5_Sn and Au-Sn grains.

### 3.3. Temperature Distribution Simulation during Bonding

In conventional bonding processes, identical temperatures are applied at the upper and bottom ends of a chip using a heating block. Heat spreads from both ends toward the center, which is referred to as isothermal bonding. After a certain period, a steady state is attained. Temperature gradient bonding (TGB) involves applying different temperatures at the two ends of bonded chips. To evaluate whether a temperature gradient exists in the TGB bonding process in our study, finite element simulation was conducted. The aim was to ascertain whether the temperature in the bonding surface remains below the melting point of Sn after reaching the steady state. Through this, we can determinate whether the Au-Sn diffusion process experiences solid-state diffusion or liquid-state diffusion.

**Figure 8:**
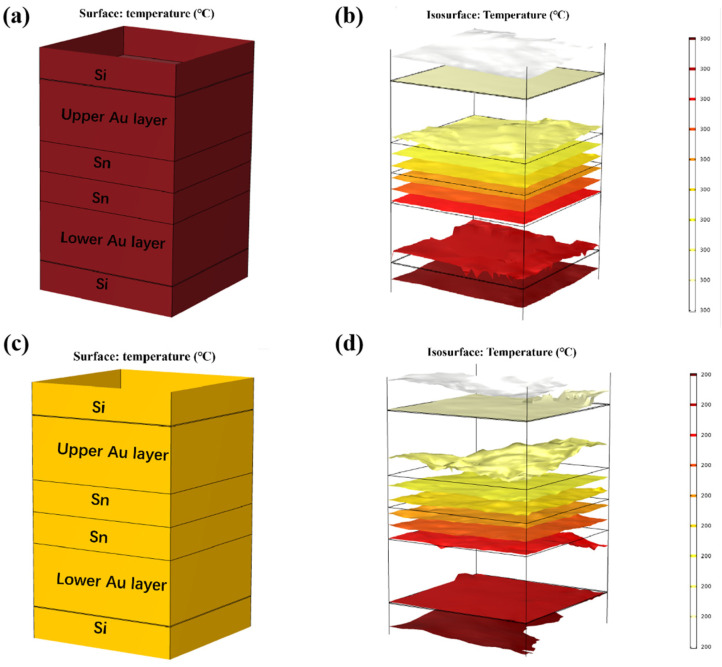
Thermal diffusion simulation of isothermal bonding at 300 °C: (**a**) temperature distribution diagram; (**b**) isotherm distribution diagram. Thermal diffusion simulation of isothermal bonding at 200 °C: (**c**) temperature distribution diagram; (**d**) isotherm distribution diagram.

Thermal diffusion simulations of isothermal bonding and TGB bonding were all conducted. The material parameters are shown in Table 1. First, isothermal bonding simulation was designed, as below. Considering that the Au-Sn bonding temperature typically exceeds 280 °C, the temperatures of the top and bottom heating blocks were set to 300 °C and 200 °C, as shown in Figure 8. The ambient temperature was set to 25 °C. The results, depicted in Figure 8a–d, revealed that the temperature within the Au/Sn bumps reached the thermal equilibrium after 10 min, yielding overall temperatures of 300 °C and 200 °C, respectively.

**Figure 9:**
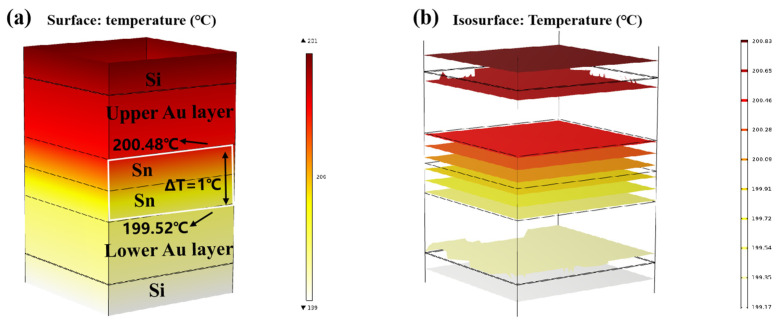
Finite element simulation of TGB thermal diffusion: (**a**) temperature distribution diagram; (**b**) isotherm distribution diagram.

Finite element simulation was carried out on the temperature gradient bonding (TGB). The top heating block was maintained at 250 °C, the bottom heating block was set at 150 °C, and the ambient temperature was set at 25 °C. As demonstrated in Figure 9a,b, thermal equilibrium was achieved within 0.01 s of heating initiation. This is attributed to the high thermal conductivity of Si, and the thin bonding metalayer totaling less than 13 µm. It also showed that the temperature within the Sn bump was around 200 °C, which was lower than the melting point of Sn. This finding confirms that the Au-Sn diffusion in the bonding process referred to the solid-state diffusion process. Moreover, the temperature gradient at the two ends of Sn layer was 1 °C. Divided by the Sn layer thickness of 4 µm, the temperature gradient was 2500 °C/cm. Existing studies suggest that the temperature gradient of 400 °C/cm is adequate to drive the atomic diffusion of Cu atoms through metal Sn [22]. An Au atom size (134) was a little larger than Cu (117) with the same lattice structure. Thus, the threshold to drive the atomic diffusion of Au atoms through metal Sn would be a little larger than that for Cu. In our study, the temperature gradient of 2500 °C/cm significantly surpassed the threshold, and thus it can drive Au atom diffusion through metal Sn. So the low-temperature Au-Sn bonding by TGB was confirmed due to the solid-state diffusion driven by the high temperature gradient in this study.

### 3.4. Au-Sn Bonding Interface under High-Temperature Annealing

**Figure 10:**
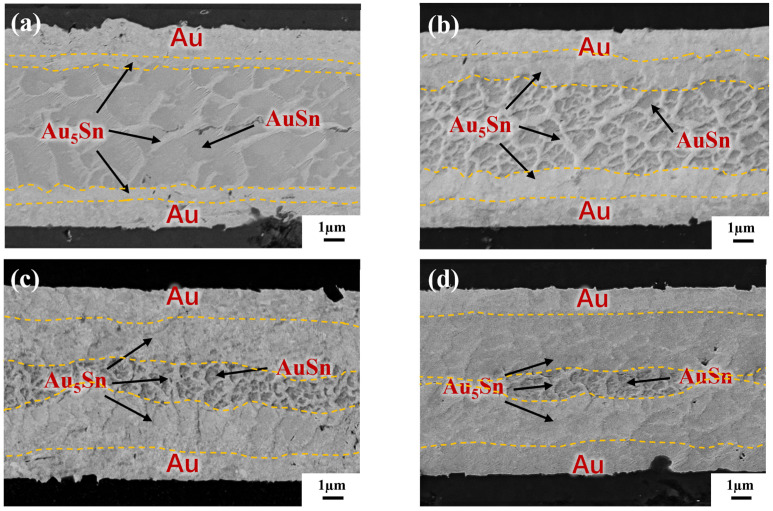
IMC evolution by TGB under different annealing times: (**a**) as-bonded (at the bonding temperature of 150 °C–250 °C with a bonding pressure of 10 MPa and bonding time of 10 min); (**b**) annealing for 30 min; (**c**) annealing for 60 min; and (**d**) annealing for 120 min.

To reveal the IMC evolution, the annealing test was carried out on the blanket Au-Sn-Au without a bump in the gradient bonding. Figure 10 separately illustrates the cross-section observation of the as-bonded samples and samples subjected to varying annealing times. In Figure 10a, it was revealed that two phases, Au_5_Sn and Au-Sn, are formed for as-bonded samples. The Au_5_Sn phase evenly distributes in the upper and lower interconnections within the reaction zone. The IMC morphology takes a like-dendritic form. The Au-Sn phase is enveloped by the Au_5_Sn phase. Figure 10b illustrates the bonding interface after 30 min annealing. At this stage, Sn is completely depleted and transformed into two phases: Au_5_Sn and Au-Sn. It is noticeable that Au-Sn starts to decrease in size with there is an increase in surrounding Au_5_Sn. The reason for this is due to the fact that long-time annealing causes more Au to react with Au-Sn, and more Au_5_Sn occurs. The continuous emergence of Au_5_Sn comes at the expense of Au and Au-Sn consumption [13], progressively reducing the Au-Sn grains. Simultaneously, the IMC thickness in the center with Au-Sn surrounded by Au_5_Sn becomes smaller. Figure 10c further convinces this assumption. After 60 min annealing, the Au-Sn size continues shrinking. The IMC interface including both Au-Sn and Au_5_Sn becomes smaller, which is hard to discern. Only a more pronounced IMC interface with a thickness of approximately 1 µm in the center was found. After 120 min of annealing treatment, as shown in Figure 10d, the conspicuous IMC interface almost disappears, leaving a large area of Au_5_Sn and Au.

## 4. Conclusions

Based on the findings and insights garnered in this study, the following conclusions and design guidelines can be drawn:

(1) The systematic optimization of Au-Sn bonding parameters, encompassing the bonding temperature, bonding time, and bonding pressure, leads to an optimized shear strength of 23.898 MPa. The corresponding bonding parameters contains gradient temperatures of 150 °C/250 °C and a bonding pressure of 10 MPa for 10 min in a HCOOH environment. IMC observation proves the high bonding strength comes from the thick IMC.
(2) Finite element simulations validated that the Au-Sn TGB bonding was solid-state bonding. It confirms when the bonding temperatures of 150 °C at the bottom and 250 °C at the top was set, the centered bonding region had the temperature around 200 °C. This resulted in a temperature gradient of 2500 °C/cm, significantly exceeding the threshold required for driving Au atoms through metal Sn. Thus, it facilitated low-temperature solid-state Au-Sn bonding.
(3) IMC evolution was explored by changing the annealing time after bonding under the optimized bonding condition. It reveals that the prolonged annealing duration results in the progressive formation of Au_5_Sn. Once Sn is depleted, Au-Sn and Au atoms combine to generate Au_5_Sn.

## Figures and Tables

**Table 1:** Thermomechanical parameters of the simulated materials.

Properties	Symbols	Au	Sn	Si	Ti
Young’s modulus	E (GPa)	79	54	150	116
Poisson’s ratio	v	0.44	0.33	0.28	0.32
Thermal conductivity	W/m·K	315	64	180	21
Density	g/cm^3^ (kg/m^3^)	19.32	7.29	2.33	4.5

## Data Availability

Data are contained within the article.
